# Xenotransplantation of interferon-gamma-pretreated clumps of a human mesenchymal stem cell/extracellular matrix complex induces mouse calvarial bone regeneration

**DOI:** 10.1186/s13287-017-0550-1

**Published:** 2017-04-26

**Authors:** Kei Takeshita, Souta Motoike, Mikihito Kajiya, Nao Komatsu, Manabu Takewaki, Kazuhisa Ouhara, Tomoyuki Iwata, Katsuhiro Takeda, Noriyoshi Mizuno, Tsuyoshi Fujita, Hidemi Kurihara

**Affiliations:** Department of Periodontal Medicine, Applied Life Sciences, Institute of Biomedical & Health Sciences, Graduate School of Biomedical & Health Sciences, Hiroshima University, 1-2-3, Kasumi, Minami-ku, Hiroshima 734-8553 Japan

**Keywords:** C-MSC, IFN-γ, IDO, Xenotransplantation

## Abstract

**Background:**

Three-dimensional cultured clumps of a mesenchymal stem cell (MSC)/extracellular matrix (ECM) complex (C-MSC) consists of cells and self-produced ECM. C-MSC can regulate the cellular function in vitro and induce successful bone regeneration using ECM as a cell scaffold. Potentiating the immunomodulatory capacity of C-MSCs, which can ameliorate the allo-specific immune response, may be helpful in developing beneficial “off-the-shelf” cell therapy for tissue regeneration. It is well reported that interferon (IFN)-γ stimulates the immunosuppressive properties of MSC via upregulation of the immunomodulatory enzyme IDO. Therefore, the aim of this study was to investigate the effect of IFN-γ on the immunomodulatory capacity of C-MSC in vitro and to test the bone regenerative activity of C-MSC or IFN-γ-pretreated C-MSC (C-MSCγ) xenografts in a mice calvarial defect model.

**Methods:**

Human bone marrow-derived MSCs were seeded at a density of 2.0 × 10^5^ cells/well into 24-well plates and cultured with growth medium supplemented with 50 μg/mL L-ascorbic acid for 4 days. To obtain C-MSC, confluent cells that had formed on the cellular sheet were scratched using a micropipette tip and were then torn off. The cellular sheet was rolled to make a round clump of cells. C-MSC was stimulated with IFN-γ and IDO expression, immunosuppressive capacity, and immunophenotype were evaluated in vitro. Moreover, C-MSC or C-MSCγ was xenotransplanted into immunocompetent or immunodeficient mice calvarial defect models without artificial scaffold, respectively.

**Results:**

IFN-γ stimulated IDO expression in C-MSC. C-MSCγ, but not C-MSC, attenuated CD3/CD28-induced T cell proliferation and its suppressive effect was reversed by an IDO inhibitor. C-MSCγ showed upregulation of HLA-DR expression, but its co-stimulatory molecule, CD86, was not detected. Xenotransplantation of C-MSCγ into immunocompetent mice calvarial defect induced bone regeneration, whereas C-MSC xenograft failed and induced T cell infiltration in the grafted area. On the other hand, both C-MSC and C-MSCγ xenotransplantation into immunodeficient mice caused bone regeneration.

**Conclusions:**

Xenotransplantation of C-MSCγ, which exerts immunomodulatory properties via the upregulation of IDO activity in vitro, may attenuate xenoreactive host immune response, and thereby induce bone regeneration in mice. Accordingly, C-MSCγ may constitute a promising novel allograft cell therapy for bone regeneration.

**Electronic supplementary material:**

The online version of this article (doi:10.1186/s13287-017-0550-1) contains supplementary material, which is available to authorized users.

## Background

Mesenchymal stem cells (MSCs) are currently the most well-studied cells for bone regenerative cell therapy because of their self-renewing property and multipotency [1]. In particular, bone marrow-derived MSCs have attracted medical and scientific attention as a preferable cellular source for bone regeneration, both in basic studies and in clinical practice [2]. It is well accepted the autologous implantation of bone marrow-derived MSCs induces bone formation at the sites of defects. However, in order to apply bone marrow-derived MSCs to established bone regenerative medicine, there still remain problems to be overcome.

One of the obstacles is attributed to patient age and condition. Previous studies revealed that aging disrupts the self-renewal ability and functionality of MSCs [3, 4]. Accordingly, it is hard for the aged patients to acquire sufficient numbers of functional MSCs for bone regenerative therapy. Moreover, the same is true for patients with bone marrow disorders [5]. The development of MSCs allograft therapy, which can supply enough functional cells stably from healthy donors, has been anticipated to overcome these problems. Indeed, since it was reported that MSCs show low immunogenicity and high immunomodulatory properties [6] in vitro, several preclinical and some clinical MSCs allograft studies have been conducted to investigate their efficacy and safety. As a result, some studies demonstrated that the application of allogenic MSCs is safe [7, 8], although others presented evidence that MSCs provoke alloimmunity and facilitate graft rejection [9–11]. Taken together, MSC utilization is still controversial and future studies are on demand to understand under which conditions MSCs can become immunogenic or not.

Very recently, we generated clumps of an MSC/extracellular matrix (ECM) complex (C-MSC), which consisted of cells and self-produced ECM [12]. C-MSC had good handleability and can be transplanted into bony lesions without artificial scaffold. These facts suggested that the application of C-MSC may be promising for tissue engineering therapy because no usage of artificial scaffold can dissolve the problems associated with biodegradability as well as undesirable host inflammatory and immunological reactions. Indeed, recent studies also reported the effectiveness of the scaffold-free fabrication of stem cell constructs for bone regenerative therapy [13, 14]. More importantly, we have discovered that the implantation of C-MSCs, cultured with osteoinductive medium in vitro, exerts more effective bone regenerative properties in a rat calvarial defect model [12]. This finding implied that C-MSCs cellular function can be regulated in vitro before transplantation. In other words, immunomodulatory properties of C-MSCs can be upregulated to establish effective allograft C-MSCs for tissue engineering therapy.

In this decade, it has been clearly revealed that interferon-gamma (INF-γ) enhances the immunosuppressive properties of MSCs [15]. Krampera et al., initially demonstrated that IFN-γ-pretreated MSC (MSCγ) attenuated T cell proliferation [16] and its molecular mechanism was due to the tryptophan catabolizing enzyme indoleamine 2,3-dioxygenease (IDO), which is well known to suppress T cell responses to prevent allogenic fetal rejection [17]. Other studies reported the therapeutic efficacy of MSCγ in allotransplantation in vivo [18, 19]. In addition, an allogenic MSC infusion abrogated kidney allograft rejection in a mice model by its IDO activity [20]. These studies suggested that MSCγ exert immunosuppressive capacity due to its highly upregulated IDO expression, thereby inhibiting allograft rejection.

Based on these accumulating lines of evidence, we hypothesized that IFN-γ treatment could also upregulate the immunosuppressive properties of C-MSC by increasing IDO expression and such C-MSCγ can be applicable for allograft bone regenerative therapy because of the highly regulated immunomodulatory function and no risk associated with inflammatory reactions to an artificial scaffold. To pursue this tentative hypothesis, we investigated the effect of IFN-γ on human C-MSC’s IDO expression level, immunomodulatory property and immunogenicity in vitro. Moreover, the bone regenerative capacity of human C-MSCγ xenotransplantation was tested in a mouse calvarial defect model.

## Methods

### Human C-MSC preparation and culture

**Fig. 1:**
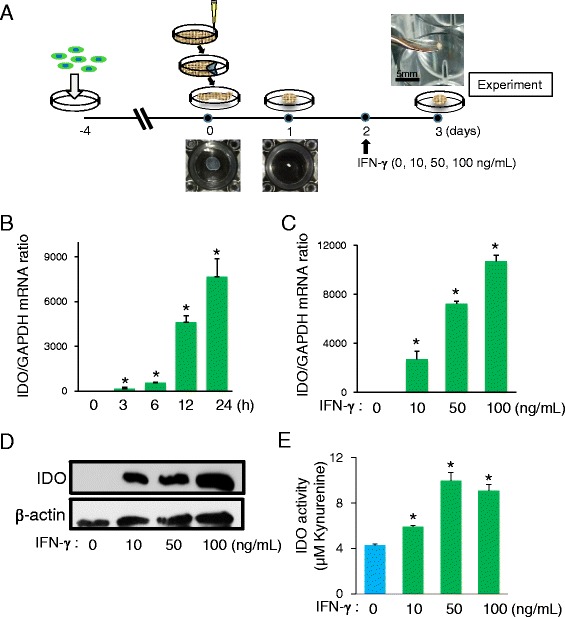
IFN-γ stimulates IDO activity in C-MSC. **a** Study design for the in vitro experiment to generate C-MSC and IFN-γ-pretreated C-MSC. C-MSCs were cultured with or without various doses of IFN-γ as shown in the schematic figure until the end of the culture period. **b** Time course study. C-MSCs were exposed to IFN-γ (10 ng/mL) for the indicated time periods. **c**-**e** Dose course study. C-MSCs were treated with or without various doses of IFN-γ (10, 50, or 100 ng/mL) for 24 h. **b** and **c** The plot shows the ratio of *IDO* mRNA to *GAPDH* mRNA. Values represent means ± S.D. of four cultures. **d** Cell lysates were collected and subjected to immunoblotting for IDO and β-actin expression. **e** The level of kynurenine in the culture supernatant was measured to monitor IDO activity in C-MSC, as described in the “Methods” section. Values represent means ± S.D. of four cultures. ^*^ *p* < 0.05, ^**^ *p* < 0.01: values differ significantly (*t* test). Similar results were obtained from three experiments. *GAPDH* glyceraldehyde-3-phosphate dehydrogenase, *IDO* indoleamine 2,3-dioxygenease, *IFN* interferon

Human bone marrow-derived MSCs (MSC-R41 and MSC-R52) were obtained from RIKEN BioResource Center (Ibaragi, Tsukuba, Japan). The cells were maintained in Dulbecco’s modified Eagle’s medium (DMEM, Sigma-Aldrich, Steinheim, Germany) supplemented with 10% fetal bovine serum (FBS, Hyclone, Logan, UT, USA), 100 U/mL penicillin (Sigma-Aldrich), and 100 μg/mL streptomycin (Sigma-Aldrich), and then C-MSCs were prepared as previously reported with minor modifications [12]. Briefly, MSCs were seeded at a density of 2.0 × 10^5^ cells/well into 24-well plates (Corning, Corning, NY, USA) and cultured with high-glucose DMEM (Sigma-Aldrich) supplemented with 10% fetal FBS, 100 U/mL penicillin, 100 μg/mL streptomycin, and 50 μg/ml L-ascorbic acid (Sigma-Aldrich) for 4 days. To obtain C-MSCs, confluent cells that had formed on the cellular sheet, consisting of the ECM produced by the MSCs themselves, were scratched using a micropipette tip and then torn off. The MSC/ECM complex was detached from the bottom of the plate in a sheet shape and rolled to make a round clump of cells. After a 1-day incubation, 1–1.5-mm-diameter C-MSCs were obtained (Fig. 1a). These were transferred into a 24-well ultra-low-binding plate (Corning) and maintained in high-glucose DMEM (Sigma-Aldrich) supplemented with 10% fetal FBS, 100 U/mL penicillin, and 100 μg/mL streptomycin (growth medium). After 2 days’ incubation in growth medium, C-MSCs were exposed to 0 to 100 ng/mL of IFN-γ (Peprotech, Rocky Hill, NJ, USA) for various periods (Fig. 1a).

### Real-time polymerase chain reaction

Total RNA from each cultured C-MSC was extracted using RNA-iso® (Takara, Otsu, Japan) and quantified by spectrometry at 260 and 280 nm. First-strand cDNA was synthesized with 1 μg of total RNA extract in a total volume of 20 μL using ReverTraAce (Toyobo, Osaka, Japan). *IDO*, osteopontin (*OPN*), alkaline phosphatase (*ALPase*), bone morphogenic protein (*BMP*)*-2*, osteocalcin (*OC*), and glyceraldehyde-3-phosphate dehydrogenase (*GAPDH*) mRNA expression levels were quantified by real-time polymerase chain reaction (PCR). *GAPDH* was used as the internal control. Real-time PCR was performed on a Lightcycler system using SYBR green (Roche Applied Science, Mannheim, Germany). The sense and antisense primers used to detect the mRNA of *IDO, OPN*, *ALPase*, *BMP-2, OC*, and *GAPHD* are listed in Table 1.

**Table 1:** Sense primers and antisense primers for real-time PCR

Target gene		Primer sequence
*IDO*	Forward	5′-CAAAGGTCATGGAGATGTCC-3′
	Reverse	5′-CCACCAATAGAGAGACCAGG-3′
*ALPase*	Forward	5′-GCGGTGAACGAGAGAATG-3′
	Reverse	5′-CGTAGTTCTGCTCGTGCAC-3′
*BMP-2*	Forward	5′-CTGTATCGCAGGCACTCA-3′
	Reverse	5′-CTCCGTGGGGATAGAACTT-3′
*OC*	Forward	5′-GCAGCGAGGTAGTGAAGAGAC-3′
	Reverse	5′-GGTCAGCCAACTCGTCACAG-3′
*OPN*	Forward	5′-GATGGCCGAGGTGATAGTGT-3′
	Reverse	5′-CCATTCAACTCCTCGCTTTC-3′
*GAPDH*	Forward	5′-AACGTGTCAGTGGTGGACCTG-3′
	Reverse	5′-AGTGGGTGTCGCTGTTGAAGT-3′

*ALPase* alkaline phosphatase, *BMP* bone morphogenic protein, *GAPDH* glyceraldehyde-3-phosphate dehydrogenase*, IDO* indoleamine 2,3-dioxygenease, *OC* osteocalcin, *OPN* osteopontin

### Immunoblotting

The C-MSCs were lysed in buffer containing 25 mM Tris-HCl (pH 7.4), 150 mM NaCl, 5 mM EDTA (pH 8.0), 0.1% SDS, 1% NP-40, 10% glycerol, and 1% (v/v) Triton X-100 [21]. The cell lysates were subjected to ultrasonic treatment for 8 s on ice. Proteins in the lysates were separated using SDS-PAGE (12% gel) and were electrophoretically transferred to a nitrocellulose (NC) membrane (Bio-Rad Laboratories, Hercules, CA, USA). The NC membranes were blocked for 1 h with 5% skim milk, followed by reaction with a rabbit anti-human IDO antibody (clone EPR1230Y, Abcam, Cambridge, MA, USA; 1:1000) or a mouse anti-β-actin antibody (Sigma-Aldrich, 1:2000) at 4 °C overnight. After extensive washes, the NC membrane was incubated for 1 h with peroxidase-conjugated donkey anti-rabbit or anti-mouse IgG antibody (Jackson ImmunoResearch, West Grove, PA, USA; 1:5000) at room temperature. The localization of specific antibodies that deposited to the molecule of interest on the NC membrane was detected using ECL Plus Western blotting detection reagents (GE Healthcare, Little Chalfont, UK).

### IDO activity assay

Since kynurenine is the product of IDO-dependent catabolism of tryptophan, the biological activity of IDO was evaluated by monitoring the level of kynurenine in C-MSC culture. One hundred microliters of culture supernatant was mixed with 50 μL of 30% trichloratic acid (Sigma-Aldrich), vortexed, and centrifuged at 10,000 × g for 5 min. Then, 75 μL of the supernatant was added to an equal volume of Ehrlich reagent (100 mg p–dimethylbenzaldehyde (Sigma-Aldrich) in 5 mL glacial acetic acid) in a 96-well plate and incubated at room temperature for 10 min. The absorbance at 492 nm was determined. The concentration of kynurenine was quantified using a standard curve generated from defined kynurenine (Sigma-Aldrich) concentrations (0–150 μM).

### Isolation of peripheral blood mononuclear cells

Peripheral blood mononuclear cells (PBMCs) were collected from healthy volunteers under informed consent agreement. The mononuclear cell fraction was separated from blood by gradient centrifugation using Histopaque-1077 (Sigma-Aldrich) and incubated in Roswell Park Memorial Institute (RPMI) 1640 supplemented with 10% FBS, 2 mM L-glutamine (Invitrogen, Carlsbad, CA, USA), 0.05 mM 2-mercaptoethanol, 100 U/mL penicillin, and 100 μg/mL streptomycin (RPMI medium).

### T cell proliferation assay

**Fig. 2:**
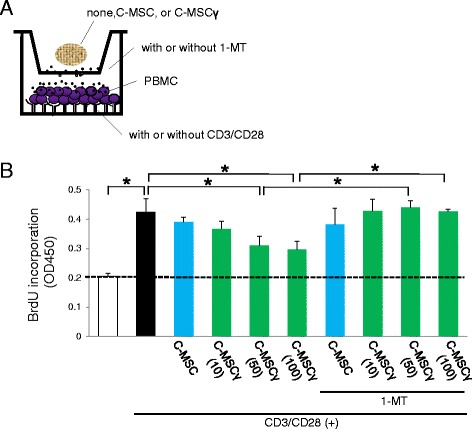
C-MSCγ attenuates T cell proliferation via its IDO activity. **a** Schematic figure of the co-culture system. Transwell culture plates with 0.4 μm pores were used. PBMCs were cultured in the bottom compartment of plates that were pre-coated with or without anti-human CD3 (5 μg/mL) and CD28 antibodies (2 μg/mL) to stimulate T lymphocyte proliferation. A C-MSC or C-MSCγ (10, 50, 100) was set in the upper chamber in the presence or absence of an IDO inhibitor, 1-MT (500 μM). They were co-cultured for 72 h in RMPI medium. **b** BrdU was added to the medium 3 h before the end of the incubation. Then, incorporated BrdU was quantified by measuring the optical density at a wavelength of 450 nm (OD450) using an ELISA kit system. Values represent means ± S.D. of four cultures. ^*^ *p* < 0.05: values differ significantly (*t* test). Similar results were obtained from three experiments. *1-MT* 1-methyltryptophan, *BrdU* bromodeoxyuridin, *CD* cluster of differentiation, *C-MSC* clumps of a mesenchymal cell/extracellular matrix complex (C-MSC) cultured in growth medium for 3 days. *C-MSCγ (10, 50, or 100)* C-MSC treated with 10, 50, or 100 ng/mL IFN-γ for 24 h before the end of the culture period, *PBMCs* peripheral blood mononuclear cells

Transwell culture plates (24-well format, see Fig. 2a) with 0.4 μm pores (Corning) were employed to test the effect of C-MSC co-culture on T cell proliferation. PBMCs in RPMI medium were cultured at 1 × 10^6^ cells/well in the bottom compartment of plates that were pretreated with mouse anti-human cluster of differentiation (CD)3 (5 μg/mL, BD Biosciences (BD), Franklin Lakes, NJ, USA) and CD28 antibodies (2 μg/mL, BD) to stimulate T lymphocytes. C-MSCs, pretreated with or without IFN-γ (10, 50, 100 ng/mL) in growth medium for 24 h, were transferred into the upper chamber of the Transwell system in the presence or absence of IDO inhibitor, 1-methyltryptophan (1-MT) (500 μM, Sigma-Aldrich). T cell proliferation was determined 3 days later by measuring the incorporation of bromodeoxyuridine (BrdU) using a cell proliferation ELISA kit (Roche, Basel, Switzerland), in accordance with the manufacturer’s instructions.

### Flow cytometric analysis

C-MSC or C-MSC pretreated with 50 ng/mL of IFN-γ for 24 h (C-MSCγ (50)) was dissociated using gentleMACS Dissociator (Milteny Biotech, Bergish Gladbach, Germany). The dissected samples were filtered through sterile 70-μm nylon cell strainers (BD) to obtain cell suspensions. The cells were then incubated with a mouse monoclonal anti-human CD90 IgG antibody (BD; 5E10), mouse monoclonal anti-human CD73 IgG antibody (BD; AD2), mouse monoclonal anti-human CD105 IgG antibody (Immunotools, Friesoythe, Germany; MEM-226), mouse monoclonal anti-CD34 IgG antibody (BD; 8G12), mouse monoclonal anti-CD45 IgG antibody (BD; 2D1), mouse anti-human human leukocyte antigen (HLA)-DR) IgG antibody (BD; G46-6), or mouse monoclonal anti-CD86 IgG antibody (BD; FUN-1) for 1 h at room temperature. The cells were then incubated with a fluorescein isothiocyanate (FITC)-conjugated goat anti-mouse-IgG antibody (Vector Laboratories Inc., Burlingame, CA, USA), for 30 min at room temperature. The expression profile of each molecule was determined using a FACScan flow cytometer (BD) with Cell Quest software (BD).

### Histological and immunofluorescence analysis of C-MSCs

C-MSC or C-MSC pretreated with 50 ng/mL of IFN-γ for 24 h (C-MSCγ (50)) was fixed with 1% paraformaldehyde and embedded in paraffin. Five-micrometer-thick serial sections were prepared. The samples were then stained with hematoxylin and eosin (H&E) and observed using a light microscope. Regarding immunofluorescence analysis, the fixed samples were embedded in Tissue-Tek OTC compound (Sakura, Torrance, CA, USA) and 20-μm-thick sections were cut by using a cryostat. The sections were washed with PBS and then nonspecific binding was blocked with 1% BSA/0.1% Triton-X/PBS blocking solution. These sections were treated with a rabbit anti-human type I collagen polyclonal IgG (Abcam 1:500) or rabbit anti-human IDO antibody (clone EPR1230Y, Abcam, 1:1000) at 4 °C overnight. After washing three times with PBS for 5 min, samples were incubated for 1 h with an Alexa Fluor 488® goat anti-rabbit IgG antibody (1:200; Invitrogen) at room temperature. Nuclei were counterstained with 4′,6-diamidino-2-phenylindole (DAPI) (5 μg/mL; Invitrogen). After rinsing the samples with PBS, fluorescence signals were detected using the Zeiss LSM 510 laser scanning confocal microscope (Zeiss MicroImaging, Inc., Thornwood, NY, USA).

### Surgical procedures

Male C57BL/6j or NOD/SCID mice (6 to 8 weeks old) (Charles River Laboratories Japan, Yokohama, Japan) were employed as a calvarial defect model after approval had been obtained from the Animal Care Committee of Hiroshima University. Surgery was performed under general anesthesia with an intraperitoneal injection of 20% ethyl carbamate (30 mg/kg body weight). The skin at the surgical site was shaved and disinfected, and a sagittal skin incision was made from the occipital to the frontal bone. The skin flap, including the periosteum, was then dissected and elevated. Avoiding the cranial suture, calvarial defects of 1.6 mm diameter were created in the parietal bone. C-MSC cultured in growth medium for 3 days was transplanted into the defect with no artificial scaffold. In addition, C-MSC pretreated with 50 ng/mL of IFN-γ for 24 h (C-MSCγ (50)) was also grafted. A no implant group was included as a control (n = 4/group). The skin incision was then closed using 4-0 silk sutures.

### Micro-computed tomography (CT) analysis

Mice were anesthetized and the cranial region was imaged using a SkyScan1176 in vivo micro-CT (Bruker, Billerica, MA, USA). Three-dimensional reconstructions were generated using CTVOX software (Bruker). The volume of newly formed bone inside the bone defect was determined using CT-An software (Bruker).

### Tissue preparation and histological analysis

Mice were sacrificed 7 or 28 days after surgery. Calvarial bones were harvested, fixed with 4% paraformaldehyde overnight, and decalcified with 10% ethylenediaminetetraacetic acid (pH 7.4) for 14 days. After decalcification, the samples were dehydrated through a graded ethanol series, cleared with xylene, and embedded in paraffin. Serial sections (5 μm) were cut in the frontal plane. These sections, representing the central portion of the bone defect, were stained with H&E and observed using a light microscope. To detect the mouse CD3 expression in the tissue, immunofluorescence analysis was performed. Briefly, the serial sections (20 μm) were blocked with 1% BSA/0.1% Triton-X/PBS blocking solution at room temperature for 30 min. These sections were then incubated with rabbit anti-mouse CD3 polyclonal IgG (Abcam; 1:500) at 4 °C overnight. After washing three times with PBS for 5 min, samples were incubated for 1 h with an Alexa Fluor 488® goat anti-rabbit IgG antibody (1:200; Invitrogen) at room temperature. Nuclei were counterstained with DAPI (5 μg/mL; Invitrogen). Fluorescence signals were detected using the Zeiss LSM 510 laser scanning confocal microscope.

### Statistical analysis

Data for in vitro studies were analyzed using Student’s *t* test. Animal studies were analyzed by Mann-Whitney *U* test. Values of *p* < 0.05 or *p* < 0.01 were considered significant.

## Results

### IFN-γ facilitates IDO expression in C-MSC to upregulate its immunomodulatory property

The study design for the experiment to generate C-MSCs is shown in Fig. 1a. Since it is well accepted that IFN-γ stimulates IDO expression level in a conventional two-dimensional culture of MSCs [15], we investigated whether its expression level in three-dimensional culture C-MSC was also elevated by IFN-γ treatment. IFN-γ facilitated the expression of *IDO* mRNA in C-MSC in both a time-dependent and dose-dependent manner (Fig.1b and c). Consistent with this finding, IDO protein production and its enzymatic activity in C-MSC were also apparently upregulated by IFN-γ stimulation (Fig. 1d and e). Then, it was examined whether C-MSCγ can exert immunomodulatory activity through IDO activity, C-MSC-PBMC co-culture system was employed (Fig. 2a). T lymphocyte stimulants, CD3/CD28 IgG treatment significantly increased PBMCs proliferation and co-culture with C-MSC did not affect its elevation (Fig. 2b). IFN-γ 50 or 100 ng/mL pretreated C-MSC, i.e., C-MSCγ (50) and C-MSCγ (100), attenuated CD3/CD28-induced PBMC proliferation, though C-MSCγ (10) (C-MSC pretreated with 10 ng/mL of IFN-γ) failed (Fig. 2b). In addition, in the presence of an IDO inhibitor, 1-MT, the inhibitory effects of C-MSCγ (50) and C-MSCγ (100) on PBMC proliferation were clearly diminished (Fig. 2b). These findings suggested that 50 or 100 ng/mL of IFN-γ stimulates IDO expression in C-MSC, which plays a role in its immunomodulatory property.

### A high concentration of IFN-γ decreases osteogenic markers in C-MSC

**Fig. 3:**
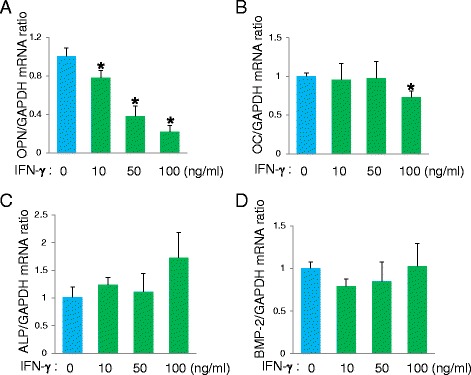
A high concentration of IFN-γ decreases bone-related protein mRNA expression in C-MSC. **a**-**d** C-MSCs were treated with or without various doses of IFN-γ (10, 50, 100 ng/mL) for 24 h. The plot shows the ratio of *OPN, OC, ALPase,* and *BMP-2* mRNAs to *GAPDH* mRNA. Values represent means ± S.D. of four cultures. Values represent means ± S.D. of four cultures. ^*^ *p* < 0.05, ^**^ *p* < 0.01: values differ significantly (*t* test). Similar results were obtained from three experiments. *ALPase* alkaline phosphatase, *BMP* bone morphogenic protein, *GAPDH* glyceraldehyde-3-phosphate dehydrogenase, *IFN* interferon, *OC* osteocalcin, *OPN* osteopontin

To establish novel allograft bone regenerative cell therapy by using C-MSCγ, the effect of IFN-γ on osteogenic marker expression should be assessed. Although IFN-γ did not affect *ALP* and *BMP-2* mRNA expression levels in C-MSC (Fig. 3c and d), *OPN* mRNA expression was significantly decreased by IFN-γ treatment in a dose-dependent manner (Fig. 3a). Moreover, 100 ng/mL of IFN-γ slightly downregulated *OCN* mRNA expression in C-MSC (Fig. 3b). These findings indicated that C-MSCγ (50) (C-MSC pre-activated with 50 ng/mL of IFN-γ), which showed effective immunomodulatory property and maintained osteogenic gene expression, may be a good candidate for the allograft bone regenerative therapy. Accordingly, in the following study, we focused on C-MSCγ (50).

### Immunophenotype of C-MSCγ

**Fig. 4:**
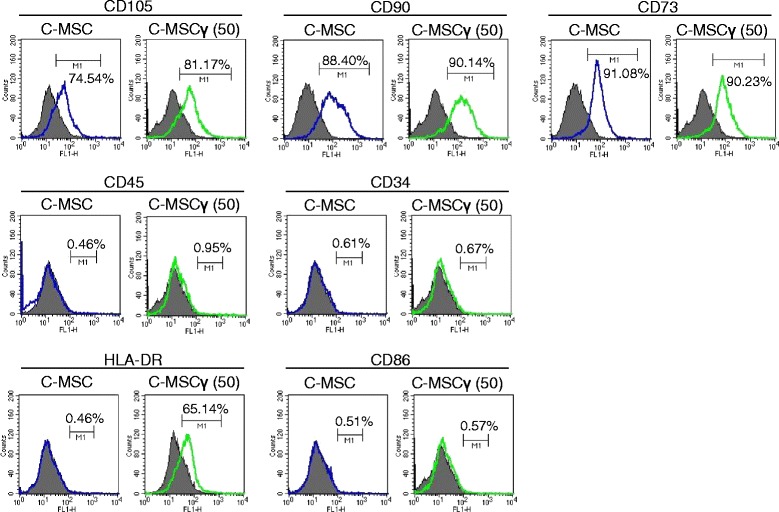
Phenotype profiles of C-MSC and C-MSCγ. Cell surface marker expression levels were monitored by flow cytometry, as described in the “Methods” section. The *open histogram* with *blue* or *green lines* indicates CD105-, CD90-, CD73-, CD34-, CD45-, HLA-DR-, or CD86-positive cells. The IgG control is shown with a *solid histogram. CD* cluster of differentiation, *C-MSC* clumps of a mesenchymal stem cell/extracellular matrix complex cultured in growth medium for 3 days, *C-MSCγ (50)* C-MSC stimulated with 50 ng/mL IFN-γ for 24 h before the end of the culture period, *HLA* human leukocyte antigens

We assessed the effect of a 1-day IFN-γ (50 ng/mL) treatment on C-MSC phenotype. Both C-MSC and C-MSCγ (50) expressed similar levels of standard MSC markers CD73, CD90, and CD105 (Fig. 4). Moreover, MSC negative markers CD34 and CD45 were not detectable (Fig. 4). C-MSC lacked HLA-DR protein expression, which is responsible for allogenic immune response, although C-MSCγ (50) showed elevation of its expression (Fig. 4). However, its co-stimulatory molecule CD86 was not detectable in both C-MSC and C-MSCγ (50) (Fig. 4).

### Xenotransplantation of human C-MSCγ, but not C-MSC, into a mouse calvarial defect model induces bone regeneration

**Fig. 5:**
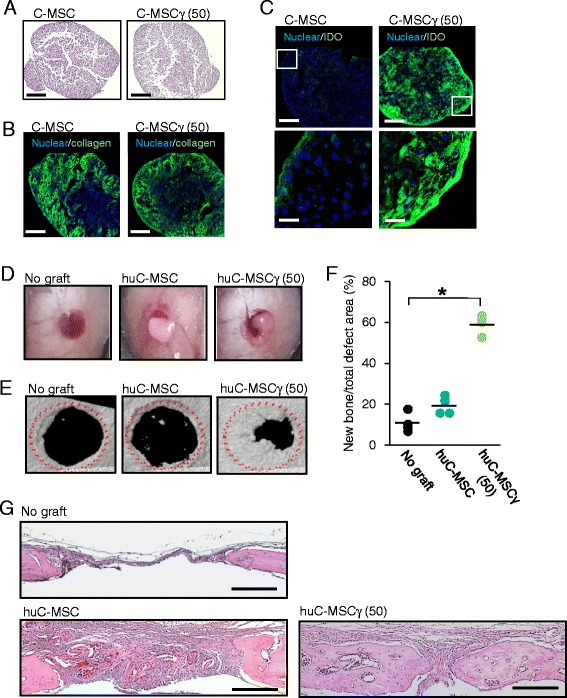
Xenotransplantation of human C-MSCγ, but not C-MSC, induced C57BL/6 mouse calvarial bone regeneration. **a** H&E staining of C-MSC or C-MSCγ. **b** Confocal immunofluorescence images of type I collagen (*green*), and nuclei (*blue*) in C-MSC or C-MSCγ. (×100: bar = 200 μm). **c** Confocal immunofluorescence images of IDO (*green*) and nuclei (*blue*) in C-MSC or C-MSCγ. *Upper panel* shows lower magnification (×100: bar = 200 μm) and the *lower panels* indicates higher magnification (×630: bar = 30 μm) C-MSC: cultured in growth medium for 3 days. C-MSCγ (50): C-MSC stimulated with 50 ng/mL of IFN-γ for 24 h before the end of the culture period. **d** Cultured human C-MSC or C-MSC*γ* (50) were transplanted into a C57BL/6 mouse calvarial defect 1.6 mm in diameter without any artificial scaffold. **e** Micro-CT images of bone regeneration by C-MSC or C-MSC*γ* (50). **f** The calvarial defect area was scanned by micro-CT on day 28. The *point* indicates the individual data and the *bar* shows the mean (n = 4/group) ^*^ *p* < 0.05: values differ significantly (*U* test). **g** All mice were sacrificed 28 days after cell transplantation and the calvarial bones were fixed. Coronal sections were obtained and stained with H&E. Lower magnification (×100) is indicated. Bar = 200 μm. *huC-MSC* xenotransplantation of human clumps of a mesenchymal stem cell/extracellular matrix complex (C-MSC) cultured in growth medium for 3 days, *huC-MSC*γ *(50)* xenotransplantation of human C-MSC treated with 50 ng/mL IFN-γ for 24 h before the end of the culture period, IDO indoleamine 2,3-dioxygenease

To assess the potency of C-MSC or C-MSCγ for human allograft bone regenerative therapy, in this present study, we examined the effect of C-MSC or C-MSCγ xenografts into a C57BL/6j mice calvarial bone defect model. Before the xenotransplantation, we assessed the shape and type I collagen, IDO expression pattern and cell viability of C-MSC or C-MSCγ (Fig. 5a-c and Additional file 1: Figure S1). TdT-mediated dUTP nick-end labeling (TUNEL) staining showed that approximately 80% of cells were viable in both C-MSC and C-MSCγ (50) (Additional file 1: Figure S1). C-MSC and C-MSCγ (50) showed similar round shapes and type I collagen expression, suggesting that IFN-γ treatment did not affect ECM production in C-MSC. Of note, consistent with the Fig. 1c, C-MSCγ (50) demonstrated a number of IDO-expressing cells, whereas few IDO-positive cells were observed in C-MSC (Fig. 5c). Then, both C-MSC and C-MSCγ (50) were directly transplanted into calvarial defects with no artificial scaffold (Fig. 5d). Compared with the no graft group, the xenotransplantation of C-MSCγ (50), but not C-MSC, clearly induced bone regeneration in the lesion area (Fig. 5e and f). To investigate whether the hard tissue observed in micro-CT images was mature newly formed bone, H&E staining was conducted. As a result, lamellar bone was observed in the C-MSCγ (50) xenografted group, whereas the implantation of C-MSC group showed only granulation-like tissue (Fig. 5g).

### C-MSCγ inhibits mouse T cell xenoreactivity in grafted regions

**Fig. 6:**
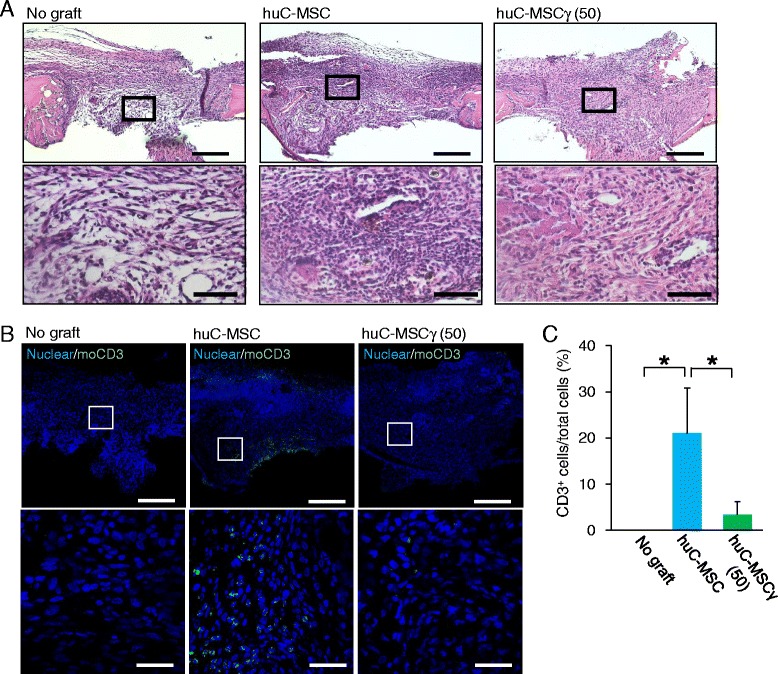
Human C-MSC but not C-MSCγ xenograft causes inflammatory cell infiltration in C57BL/6 mouse calvarial bone defect. Cultured human C-MSC or C-MSC*γ* (50) were transplanted into a C57BL/6 mouse calvarial defect 1.6 mm in diameter. All mice were sacrificed 7 days after cell transplantation and the calvarial bones were fixed. Coronal serial sections were obtained and H&E staining (**a**) and immunofluorescence staining of mouse CD3 (**b**) were performed. The *upper panels* show lower magnification ((**a** and **b**) × 100: bar = 200 μm) and the *lower panels* indicates higher magnification ((**a**) × 400: bar = 50 μm or (**b**) × 630: bar = 30 μm). **c** Four arbitrary views in the bone defect region from each group were used for counting of CD3-positive cells. Results are expressed as means ± S.D. of the four views tested for each group. ^*^ *p* < 0.05: values differ significantly (*U* test). *CD* cluster of differentiation, *huC-MSC* xenotransplantation of human clumps of a mesenchymal stem cell/extracellular matrix complex (C-MSC) cultured in growth medium for 3 days, *huC-MSC*γ *(50)* xenotransplantation of human C-MSC treated with 50 ng/mL IFN-γ for 24 h before the end of the culture period

Based on the findings in Fig. 5, we hypothesized that xenograft of human C-MSC induced mice T cell immune response, which may be associated with a failure of bone regeneration, whereas C-MSCγ attenuates such xenoreactive T cell response. To investigate this hypothesis we observed the early-stage transplantation of C-MSCs by histological and immunofluorescence analysis. Seven days after surgery, H&E staining showed only sparse connective tissue in the no graft group (Fig. 6a). In the C-MSC grafted group, thicker fibrotic tissue was observed. Additionally, remarkable inflammatory cells infiltration was also found in the bony lesion area (Fig. 6a). On the other hand, C-MSCγ (50) implantation showed abundant connective tissue and few inflammatory cells (Fig. 6a). According to the confocal microscopy that stained mouse T lymphocyte-specific CD3 in the bony lesion, few or no CD3+ T cells were detected in the no graft group, although apparent CDC3+ T cells were observed in the C-MSC graft area (Fig. 6b and c). However, as we expected, C-MSCγ (50) transplantation did not induce such CD3+ T cells infiltration (Fig. 6b and c). These findings suggested that C-MSCγ attenuates mice xenoreactive T cell activity in the calvarial bone defect area.

### Xenotranplantation of both human C-MSC and C-MSCγ into immunodeficient mice calvarial bone defects

**Fig. 7:**
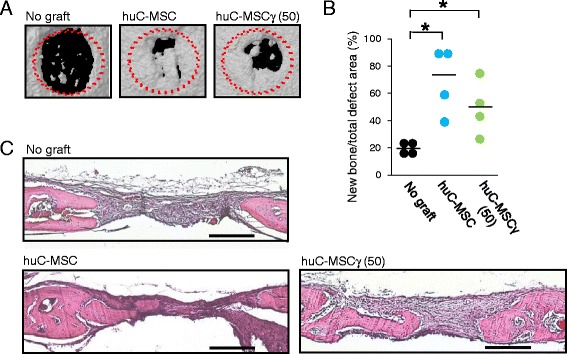
Both human C-MSC and C-MSCγ xenografts induce SCID mouse calvarial bone regeneration. Cultured human C-MSC or C-MSC*γ* (50) in vitro were transplanted into a NOD/SCID mouse calvarial defect 1.6 mm in diameter. **a** Micro-CT images of bone regeneration by C-MSC or C-MSC*γ* (50). **b** The calvarial defect area was scanned by micro-CT on day 28. The *point* indicates the individual data and the *bar* shows the mean (n = 4/group). ^*^ *p* < 0.05: values differ significantly (*U* test). **c** All mice were sacrificed 28 days after cell transplantation and the calvarial bones were fixed. Coronal sections were obtained and stained with H&E. Lower magnification (×100) is indicated. Bar = 200 μm. *huC-MSC* xenotransplantation of human clumps of a mesenchymal stem cell/extracellular matrix complex (C-MSC) cultured in growth medium for 3 days, *huC-MSC*γ *(50)* xenotransplantation of human C-MSC treated with 50 ng/mL IFN-γ for 24 h before the end of the culture period

Finally, to test whether a xenoreactive immune response is engaged in the failure of bone regeneration by C-MSC implantation, a NOD/SCID mice calvarial defect model was employed. Compared with the no graft group, both C-MSC and C-MSCγ (50) xenografts induced similar levels of mineralized tissue formation (Fig. 7a and b). Consistent with these micro-CT findings, both C-MSC and C-MSCγ (50) transplantation facilitated bone regeneration in the defect areas (Fig. 7c). These results implied that C-MSC possesses bone regenerative property identical to C-MSCγ in the absence of xenoreactive immune responses in vivo.

## Discussion

We have previously demonstrated that syngenic rat C-MSC transplantation into a rat calvarial defect model caused successful bone regeneration [12]. In this present study, human C-MSC xenograft into immunocompetent mice calvarial defect induced T cell infiltration and failed to induce bone regeneration, whereas xenotransplantation of C-MSCγ, which showed higher IDO expression level and T cell suppressive capacity in vitro, caused bone healing. On the other hand, both C-MSC and C-MSCγ xenograft into immunodeficient mice induced bone regeneration. These findings suggested that (1) C-MSC possessed bone regenerative property equivalent to that of C-MSCγ, (2) xenogenic immune response disrupted the bone healing induced by C-MSC, and (3) the immunomodulatory property of C-MSCγ was sufficient to eliminate such undesirable immune response to induce successful bone regeneration. Supporting our findings, Chuang et al., demonstrated that human MSC xenotransplantation using a poly lactic-co-glycolic acid (PLGA) scaffold into immunocompetent rat calvarial defects required immunosuppressive drugs, which can protect the grafted cells from host immune response, to induce bone regeneration [22]. These facts may imply that C-MSCγ may be applicable for clinical allogenic bone regenerative cell therapy due to their highly regulated immunomodulatory properties.

The precise underlying molecular mechanism by which C-MSCγ modulate the mouse immune response is unclear. Because we did not conduct the co-culture assay by using mouse periphery blood cells, although C-MSCγ inhibited human T cell proliferation in vitro (Fig. 2). However, IDO, which was activated in C-MSCγ, appeared to be the responsible molecular factor. Because, it is widely accepted that IFN-γ stimulates IDO expression, which plays a crucial role in immunosuppressive capacity in conventional two-dimensional cultured MSCs [23–25]. More specifically, IFN-γ-induced IDO catalyzes the conversion from tryptophan to kynurenine and the starvation of tryptophan and activated kynurenine pathway are associated with inhibition of murine T cell proliferation [26, 27]. Indeed, three-dimensional cultured C-MSC treated with IFN-γ, i.e., C-MSCγ, showed increased kynurenine production in culture supernatant (Fig. 1), suggesting the inhibitory capacity on murine immune system.

However, recently, the other molecules responsible for immunomodulatory property of MSCγ were also discovered. For example, Zhang et al. demonstrated that not only IDO but also interleukin 10 (IL-10) was increased by IFN-γ treatment in human gingival MSCs and bone marrow-derived MSCs, which was attributed to their immunosuppressive function [28]. Therefore, we investigated the effect of IFN-γ on IL-10 production in cultured C-MSC. Although IFN-γ clearly facilitated IL-10 mRNA expression level in C-MSC, the protein was not detectable in the culture supernatant (Additional file 1: Figure S1). Since C-MSC consisted of abundant self-produced ECM, cell-secreted cytokines may be trapped in the ECM. In addition, IFN-γ induced immunosuppressive property of MSCs through upregulating programmed cell death ligand 1 (PD-L1), which binds to programmed cell death 1 (PD-1) in immune cells to modulate their activation [29, 30]. It is plausible that IL-10 or PD-L1 play an immunomodulatory role in C-MSCγ transplantation. Indeed, after 7 days transplantation, C-MSC did not retain the round shape, suggesting the biological absorption of ECM. In this condition, cytokines trapped in ECM could be released into the tissue, and the grafted cells can be distributed to bind their PD-L1 to PD1 in host immune cells. Accordingly, not only IDO, but also other cytokines or cell surface proteins may be candidates for the immunomodulatory tools of C-MSCγ in this xenograft model.

MSCs are well known to express low levels of HLA-DR and its co-stimulatory molecules, which trigger the activation of allospecific T cell responses, and thereby act to escape host immune system [31]. This low immunogenicity has gathered great attention as a safe MSC allo-transplantation therapy. Considering this immunogenicity, C-MSCγ allo-graft therapy may require some circumspection. A number of studies reported that IFN-γ upregulates HLA-DR expression level in MSCs [16, 32, 33]. In agreement with these previous reports, C-MSCγ also expressed higher level of HLA-DR than that of C-MSC (Fig. 4). Since C-MSCγ lacked the expression of co-stimulatory molecule CD86 (Fig. 4), it might be expected to escape the allo-reactive host immune system. However, the immunogenicity of MSCγ is still very controversial. Some reports demonstrated that allograft of MSCγ did not cause allogenic immune rejection because of the lack of the co-stimulatory signals, including those such as CD80, CD83, and CD86 [16, 23, 33, 34]. In contrast, others clearly showed that IFN-γ increased the immunogenicity of MSCs to induce allogenic host immune response [35–37]. Although xenograft of C-MSCγ induced bone regeneration caused by the upregulated immunomodulatory property, concern remains about whether upregulated HLA-DR can be immunogenic or not. HLA-matched C-MSCγ may be safer and more reliable for clinical allograft bone regenerative therapy.

Several reports have shown that transplanted MSCs can stimulate bone repair based on its multiple regenerative abilities, which include direct cell differentiation effects [38] and on its indirect paracrine effects, such as modulation of the local inflammatory environment and stimulation of host tissue repair [39, 40]. It has been reported that a high concentration of IFN-γ inhibits osteogenesis of BMMSCs [41, 42] through upregulated IDO activity [41]. Therefore, although we have decided on 50 ng/mL IFN-γ as the appropriate dose to establish C-MSCγ for xenograft bone regenerative therapy, its direct osteoblastic differentiation ability seems to be lower than that of non-treated C-MSC. Moreover, it was reported that IFN-γ-mediated IDO expression in MSCs stimulates the differentiation of monocytes into immune-suppressive and tissue-reparative M2 macrophage [43]. Taken together, these findings may imply that the molecular mechanism of C-MSCγ in the bone-healing process may be mainly associated with an indirect paracrine effect but not direct osteogenic differentiation, although additional investigations are needed.

Very recently, Sivanathan et al. revealed that IL-17A preconditioned MSCs, i.e., MSC-17, functioned as effectively as MSCγ at suppressing T cell activation in vitro, whereas they did not increase IDO expression [44]. It is of interest to note that MSC-17, in contrast to MSCγ, did not increase HLA-DR expression, suggesting its low immunogenicity. Although the mechanism of T cell suppression by MSC-17 was unclear, they implied that cell surface molecules might be responsible based on a direct co-culture study. Accordingly, C-MSC pretreated with IL-17A, i.e., C-MSC-17 may not be an appropriate study model to investigate immunosuppressive molecules. However, C-MSC-17 might be a good candidate for allograft bone regenerative cell therapy because of their high immunomodulatory property and low immunogenicity.

For C-MSCγ tissue engineering therapy, larger bone defect cases at the clinical side still seem to be a challenge. For instance, a segmental tibial fracture with a 4 cm gap will require the transplantation of approximately 200–300 C-MSCs. It is unclear if all transplanted C-MSCs remain in the lesion area. Accordingly, in order to graft more than several hundred C-MSCs appropriately into larger damaged tissue areas, combined use of some artificial scaffold may be needed.

## Conclusions

IFN-γ treatment of C-MSC upregulated IDO expression and T cell suppressive property in vitro. Xenograft of C-MSCγ with no artificial scaffold retained elevated immunomodulatory capacity and induced bone regeneration in a mouse calvarial defect. Therefore, C-MSCγ, which can avoid the problems associated with usage of an artificial scaffold, such as biodegradability or host inflammatory reaction, may represent a novel allograft cell therapy for bone defect diseases because of its highly regulated immunomodulatory function. In addition, a future study to reduce C-MSCγ immunogenicity may lead the reliable “off-the-shelf” MSC therapy for tissue regeneration.

## Additional file

Additional file 1: Figure S1. Cell viability in C-MSC and C-MSCγ. TUNEL staining for apoptotic cells was performed using the DeadEnd™ Fluorometric TUNEL System (Promega, Madison, WI, USA). (A) Confocal immunofluorescence images of TUNEL (*green*) and nuclei (*blue*) in C-MSC or C-MSCγ. *Upper panel* shows lower magnification (bar = 200 μm) and the *lower panels* indicates higher magnification (bar = 50 μm). (B) The graphs show the percentage of TUNEL-positive apoptotic cells. Values represent means ± S.D. of three cultured C-MSCs. Similar results were obtained from three independent experiments. *C-MSC* clumps of a mesenchymal cell/extracellular matrix complex (C-MSC) cultured in growth medium for 3 days, *C-MSCγ (50)* C-MSC stimulated with 50 ng/mL of IFN-γ for 24 h, *TUNEL* TdT-mediated dUTP nick-end labeling. **Figure S2**. IFN-γ increased IL-10 mRNA expression, but not its protein production in C-MSC. (A)Time course study. C-MSCs were exposed to IFN-γ (10 ng/ml) for the indicated time. (B and C) Dose course study. C-MSCs were treated with or without various doses of IFN-γ (10, 50, 100 ng/ml) for 24 h. (A and B) IL-10 mRNA expression level was evaluated by real-time PCR as described in the “Methods” section. Sequence of primers for IL-10 was 5’-TCAAACTCACTCATGGCTTTGT-3’ (forward) and 5’-GCTGTCATCGATTTCTTCCC-3’ (reverse). The plot shows the ratio of *IL-10* mRNA to *GAPDH* mRNA. Values represent means ± S.D. of four cultures. (C) The IL-10 protein production in culture supernatant was measured by IL-10 ELISA kit (Peprotech) following the manufacturer’s instruction. Data are the means ± S.D. of four cultures. *GAPDH* glyceraldehyde-3-phosphate dehydrogenase, *IDO* indoleamine 2,3-dioxygenease, *IFN* interferon, *IL* interleukin (PDF 378 kb)

## Abbreviations

1-MT: 1-Methyltryptophan
ALPase: Alkaline phosphatase
BMP: Bone morphogenic protein
BrdU: Bromodeoxyuridin
CD: Cluster of differentiation
C-MSC: Clumps of a mesenchymal stem cell/extracellular matrix complex
C-MSCγ: Interferon-γ-pretreated C-MSC
CT: Computed tomography
DAPI: 4′,6-diamidino-2-phenylindole
DMEM: Dulbecco’s modified Eagle’s medium
ECM: Extracellular matrix
FBS: Fetal bovine serum
FITC: Fluorescein isothiocyanate
GAPDH: Glyceraldehyde-3-phosphate dehydrogenase
H&E: Hematoxylin and eosin
HLA: Human leukocyte antigens
IDO: Indoleamine 2,3-dioxygenease
IFN: Interferon
IL: Interleukin
MSC: Mesenchymal stem cell
MSCγ: IFN-γ-pretreated MSC
NC: Nitrocellulose
OC: Osteocalcin
OPN: Osteopontin
PBMCs: Peripheral blood mononuclear cells
PD-1: Programmed cell death 1
PD-L1: Programmed cell death ligand 1
PLGA: Poly lactic-co-glycolic acid
RPMI: Roswell Park Memorial Institute
TUNEL: TdT-mediated dUTP nick-end labeling
