# Supplementary material for: Xenotransplantation of interferon-gamma-pretreated clumps of a human mesenchymal stem cell/extracellular matrix complex induces mouse calvarial bone regeneration
Source: Stem Cell Res Ther. 2017 Apr 26;8:101. doi: 10.1186/s13287-017-0550-1 (PMC5406942; doi:10.1186/s13287-017-0550-1)
Supplement: Additional file 1: Figure S1. — Cell viability in C-MSC and C-MSCγ. TUNEL staining for apoptotic cells was performed using the DeadEnd™ Fluorometric TUNEL System (Promega, Madison, WI, USA). (A) Confocal immunofluorescence images of TUNEL (green) and nuclei (blue) in C-MSC or C-MSCγ. Upper panel shows lower magnification (bar = 200 μm) and the lower panels indicates higher magnification (bar = 50 μm). (B) The graphs show the percentage of TUNEL-positive apoptotic cells. Values represent means ± S.D. of three cultured C-MSCs. Similar results were obtained from three independent experiments. C-MSC clumps of a mesenchymal cell/extracellular matrix complex (C-MSC) cultured in growth medium for 3 days, C-MSCγ (50) C-MSC stimulated with 50 ng/mL of IFN-γ for 24 h, TUNEL TdT-mediated dUTP nick-end labeling. Figure S2. IFN-γ increased IL-10 mRNA expression, but not its protein production in C-MSC. (A)Time course study. C-MSCs were exposed to IFN-γ (10 ng/ml) for the indicated time. (B and C) Dose course study. C-MSCs were treated with or without various doses of IFN-γ (10, 50, 100 ng/ml) for 24 h. (A and B) IL-10 mRNA expression level was evaluated by real-time PCR as described in the “Methods” section. Sequence of primers for IL-10 was 5’-TCAAACTCACTCATGGCTTTGT-3’ (forward) and 5’-GCTGTCATCGATTTCTTCCC-3’ (reverse). The plot shows the ratio of IL-10 mRNA to GAPDH mRNA. Values represent means ± S.D. of four cultures. (C) The IL-10 protein production in culture supernatant was measured by IL-10 ELISA kit (Peprotech) following the manufacturer’s instruction. Data are the means ± S.D. of four cultures. GAPDH glyceraldehyde-3-phosphate dehydrogenase, IDO indoleamine 2,3-dioxygenease, IFN interferon, IL interleukin (PDF 378 kb) [file 13287_2017_550_MOESM1_ESM.pdf]

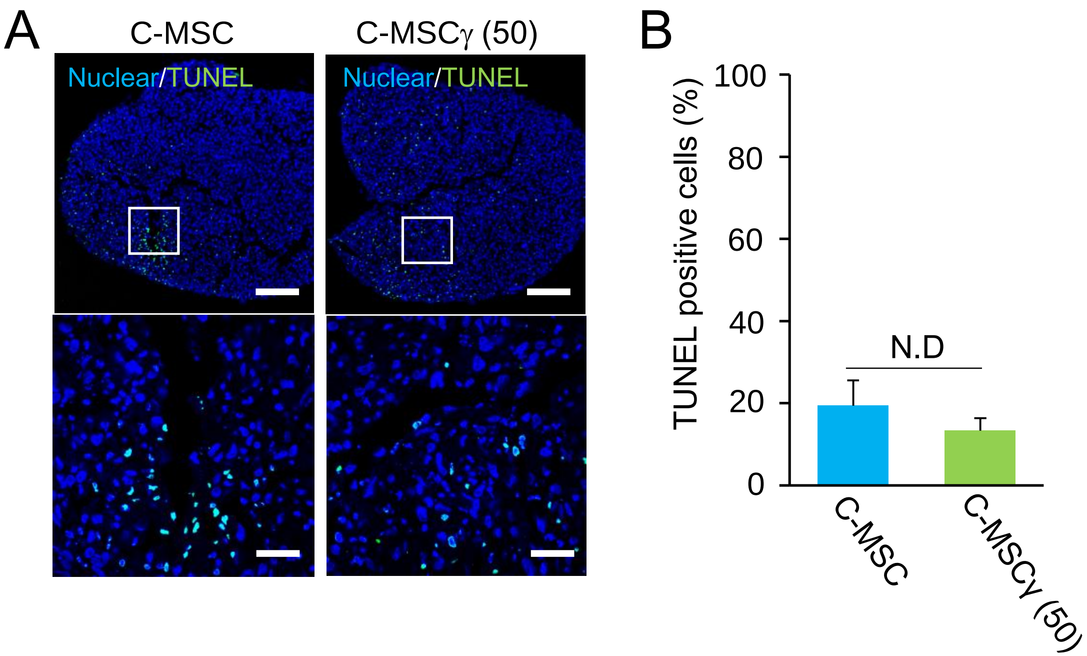


*Supplemental Fig. 1 Cell viability in C-MSC and C-MSCγ*

TUNEL staining for apoptotic cells was performed using the DeadEnd™ Fluorometric TUNEL System (Promega). (A) Confocal immunofluorescence images of TUNEL (green) and nuclei (blue) in C-MSC or C-MSCγ. Upper panel shows lower magnification (bar=200 μm) and the lower panels indicates higher magnification (bar=50 μm) (B) The graphs show the percentage of TUNEL-positive apoptotic cells. Values represent means ± S.D. of three cultured C-MSCs. Similar results were obtained from three independent experiments. C-MSC: Cultured in growth medium for 3 days. C-MSCγ (50): C-MSC stimulated with 50 ng/mL of IFN-γ for 24 h.

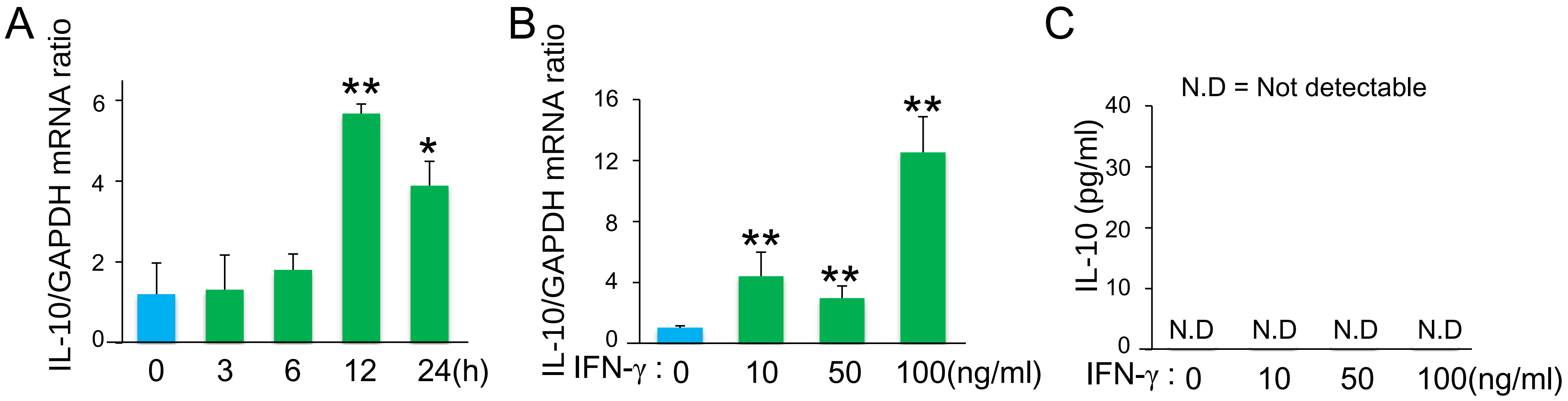


*Supplemental Fig. 2 IFN-γ increased IL-10 mRNA expression, but not its protein production in C-MSC*

(A) Time course study. C-MSCs were exposed to IFN-γ (10 ng/ml) for the indicated time. (B and C) Dose course study. C-MSCs were treated with or without various doses of IFN-γ (10, 50, 100 ng/ml) for 24 hr. (A and B) IL-10 mRNA expression level was evaluated by real-time PCR as described in the Materials and Methods section. Sequence of primers for IL-10 were 5'-TCAAACTCACTCATGGCTTTGT-3' (forward) and 5'-GCTGTCATCGATTTCTTCCC-3' (reverse). The plot shows the ratio of *IL-10* mRNA to *GAPDH* mRNA. Values represent means ± S.D. of four cultures. (C) The IL-10 protein production in culture supernatant was measured by IL-10 ELISA kit (Peprotech) following the manufacturer's instruction. Data are the means ± S.D. of four cultures.
